# Real-time qPCR for the detection of puffer fish components from *Lagocephalus* in food: *L. inermis*, *L. lagocephalus*, *L. gloveri*, *L. lunaris*, and *L. spadiceus*

**DOI:** 10.3389/fnut.2022.1068767

**Published:** 2022-12-05

**Authors:** Xinying Yin, Ranran Xing, Zhiru Li, Bing Hu, Lili Yang, Ruijie Deng, Jijuan Cao, Ying Chen

**Affiliations:** ^1^Key Laboratory of Biotechnology and Bioresources Utilization of Ministry of Education, Dalian Minzu University, Dalian, China; ^2^Healthy Food Evaluation Research Center, College of Biomass Science and Engineering, Sichuan University, Chengdu, China; ^3^Chinese Academy of Inspection and Quarantine, Beijing, China

**Keywords:** food authenticity, food adulteration, *Lagocephalus*, qPCR, puffer fish

## Abstract

Puffer fish is a type of precious high-end aquatic product, is widely popular in Asia, especially in China and Japan, even though it naturally harbors a neurotoxin known as tetrodotoxin (TTX) that is poisonous to humans and causes food poisoning. With the increasing trade demand, which frequently exceeds existing supply capacities, fostering fraudulent practices, such as adulteration of processed products with non-certified farmed wild puffer fish species. To determine the authenticity of puffer fish processed food, we developed a real-time qPCR method to detect five common puffer fish species in aquatic products: *Lagocephalus inermis*, *Lagocephalus lagocephalus*, *Lagocephalus gloveri*, *Lagocephalus lunaris*, and *Lagocephalus spadiceus*. The specificity, cross-reactivity, detection limit, efficiency, and robustness of the primers and probes created for five species of puffer fish using TaqMan technology have been determined. No cross-reactivity was detected in the DNA of non-target sample materials, and no false-positive signal was detected; the aquatic products containing 0.1% of a small amount of wild puffer fish materials without certification can be reliably tracked; the statistical *p*-value for each method’s *Ct* value was greater than 0.05. The developed qPCR method was sensitive, highly specific, robust, and reproducibility, which could be used to validate the authenticity of wild puffer fish in aquatic products sold for commercial purposes.

## Introduction

Puffer fish (Tetraodontidae) generally belongs to the genera of *dontidae*, *Tetraodontiformes*, and *Actinopterygii*. Despite its recognized potential toxicity caused by Tetrodotoxin (TTX), puffer fish is a long-standing delicacy in China, Japan, and other Asian nations, and is regarded as the “top of dishes” (1). TTX is the naturally occurring toxin harbored in puffer fish’s ovaries, liver, kidneys, eyes, and blood (2). Therefore, improper handling or accidental consumption of puffer fish can result in severe toxicity and even death. In addition, TTX is present in the muscles of a number of puffer fish, and because the TTX content in the muscles of some puffer fish is lethal, many poisoning occurrences have been caused by the consumption of processed and cooked puffer fish (3). In Japan, the preparation of puffer fish needs special training. In China, the sale of fresh puffer fish is banned. However, since 2016, the latest regulations permit *Takifugu rubripes* and *Takifugu obscurus* to be farmed by certified companies and sold after processing, with a code on the package to track the products’ origin. Since 2016, both species have become available in China’s local markets, and approximately 70% of the annual production is exported (1, 4, 5). The rapid expansion of the high-end aquatic product trade has led to an increase in demand, which is conducive to food fraud, such as incorrect labeling and the substitution of non-certified cultured puffer fish for wild puffer fish goods. In addition, because the morphological characteristics of puffer fish are highly similar (6, 7), it is difficult for inexperienced consumers to correctly identify morphologically, particularly after the fish has been processed (8). Due to their similar appearance, using the wrong species puffer fish may lead to poisoning risk to consumers (9).

Food authenticity identification technology has been developed to ensure food safety and quality control. However, processed foods have often been destroyed in their morphological features and cannot effectively identify in terms of morphology (8). To evaluate food authenticity, a significant amount of research has been conducted in recent years on omics-based food authenticity recognition technologies, including genomics, proteomics, and metabolomics. Proteomics studies the existence state and activity rules of proteins at the overall level under specific conditions, which cannot only identify protein species but also quantify proteins. Proteomics is based on protein databases for species identification, origin tracing, quality identification, and other food authenticity identification (10–13). However, protein-based methods can hardly find target protein in heat-treated foods due to the denaturation of proteins at high temperatures. The examination of metabolites based on metabolomics is primarily separated into target analysis and non-target analysis, including vibration spectrum, chromatography-mass spectrum, nuclear magnetic resonance, etc. (14). Omics-based methods have become a comprehensive solution for food fraud (15, 16).

PCR-based methods for the detection and differentiation of species have usually been applied due to their high specificity, sensitivity, and speed, including qPCR (17), digital PCR (18, 19), gene chip (20), and DNA barcode (21) which can quickly distinguish all animals and plants raw materials used in food and has attracted international attention and developed rapidly. TaqMan-based real-time quantitative polymerase chain reaction (qPCR) plays an important role in food authenticity identification. This method is highly sensitive, specific and DNA is stable at a high temperature and can be extracted in most cells. Such methods have been successfully developed to detect different materials affected by fraud. DNA-based molecular biology methods are still considered the most effective method for food authenticity identification (22). By selecting appropriate target genes based on the characteristics of gene evolution, it is possible to achieve satisfactory species and strain distinction. Even with certain biologically distinct individuals (23–26).

Here, we developed a real-time PCR method based on the TaqMan probe to identify the components of puffer fish of the genus *Lagocephalus* in food, including *Lagocephalus inermis*, *Lagocephalus lagocephalus*, *Lagocephalus gloveri*, *Lagocephalus lunaris*, and *Lagocephalus spadiceus*. This method is based on the amplification of the *cytochrome oxidase subunit I* (*COI*) gene. Due to the high variability of *COI*, it was selected to qualitatively identify the species of puffer fish of the genus *Lagocephalus*. The specificity of this method is determined by detecting cross-reactivity with other puffer fish family members and common fish species. The limit of detection (LOD) and stability of the method were evaluated.

## Materials and methods

### Materials

Complete samples of puffer fish have been identified by morphology. All puffer fish samples were provided by the Fisheries Research Institute of Fujian, including *Lagocephalus inermis*, *Lagocephalus lagocephalus*, *Lagocephalus gloveri*, *Lagocephalus lunaris*, *Lagocephalus spadiceus*, *Takifugu vermicularis*, *Takifugu fasciatus*, *Takifugu xanthopterus*, *Takifugu bimaculatus*, *Takifugu flavidus*, *Takifugu rubripes*, *Takifugu oblongus*, *Takifugu alboplumbeus*. Other fish samples used for the specificity test have also been identified in morphology, including *Limanda aspera*, *Verasper variegatus*, *Verasper moseri*, *Platichthys stellatus*, *Paralichthys lethostigma*, *Oncorhynchus gorbuscha*, *Gadus macrocephalus*, *Sebastes schlegelii*, *Trachurus japonicus* were obtained from Dalian Tianzheng Industrial Co., Ltd (Dalian, China). All fish materials information was listed in Supplementary Tables 1, 2. DNA oligonucleotides were synthesized by TaKaRa (Dalian, China) and set out in Supplementary Table 3. All sequences were purified by polyacrylamide gel electrophoresis (PAGE).

### Deoxyribonucleic acid extraction

Fish meat samples were pulverized using a high-speed tissue masher (34BL99, Waring Blender dynamics Corp., New Hartford, CT, USA). Ground sample (200 mg) was taken for DNA extraction. DNA Extraction Kit (Code No. 9766, TaKaRa Co., Ltd., Dalian, China) was used according to the manufacturer’s recommendations. The operations were as follows: 10 mg sample materials were taken for low-temperature grinding by adding liquid nitrogen, then added 200 μL PBS buffer. Briefly, 200 μL VGB buffer, 20 μL proteinase K, and 1.0 μL carrier RNA were added and fully mixed in a 56°C water bath for 10 min. Then 200 μL 96–100% ethanol was added, and fully mixed. Placed the spin column on the collection tube, transferred the solution to the spin column, centrifuge at 12,000 × g for 2 min, and discarded the filtrate. Then, 500 μL RWA buffer was added to the spin column, 12,000 × g centrifuge for 1 min, and discarded the filtrate. And then, 700 μL buffer RWB was added to the spin column, 12,000 × g centrifuge for 1 min, and discard the filtrate. Repeat the previous step. Placed the spin column on the collection tube, and 12,000 × g centrifuge for 2 min. Placed the spin column in a new 1.5 ml RNase-free collection tube, and added 30–50 μL RNase-free dH_2_O, standing at room temperature for 5 min. Centrifugation at 12,000 rpm for 5 min. The products were dissolved in H_2_O.

### Sequence retrieval and analysis

Puffer fish *COI* gene sequences of mitochondrial were retrieved from the official National Center for Biotechnology Information (NCBI) database GenBank.^1^ And then, these sequences as the template used for Blast analysis. In addition, the specificity of the primer and probes was tested by Blast. MEGA 4.0 software (27) was used to perform sequence alignment to screen high variability DNA fragments, examine the specificity of primers and probes, and guarantee that the primers and probes cannot theoretically amplify genes from related species. All sequence accession numbers were listed in Supplementary Table 2.

### Quantitative polymerase chain reaction primers and probes design

Arrange the *COI* sequence of the target species and the DNA sequence of the most relevant species (such as the common puffer fish species of the genus Fugu) and screen for the fragments with the greatest variability.

Considering the impact of food processing on DNA quality, the amplification efficiency of real-time qPCR analysis was improved by designing primer pairs to amplify relatively short DNA fragments. The nucleotide sequences chosen for primer design were introduced into the program “Oligocalc” (28), and the length was optimized for the resulting “salt-adjusted” annealing temperature. Then, the annealing temperatures calculated by “Oligocalc” applying the “salt-adjusted” algorithm were used as starting values for the qPCR. Four qPCR methods were based on the TaqMan probe, modified with the reporter fluorophore, 6-carboxyfluorescein (FAM), and quencher fluorophore black hole quencher (BHQ_1) at 5’ and 3’ end, respective. *L. inermisand* and *L. lagocephalus* amplification fragment sizes were 196 bp, *L. gloveri* was 174 bp, *L. lunaris* was 150 bp, and *L. spadiceus* was 173 bp. Finally, 18SrRNA was used as the control gene to design primer and probe for detecting DNA of all sample materials to ensure no inhibitory contaminants. All primers and probes oligonucleotide sequences were listed in Supplementary Table 3 and synthesized by TaKaRa (Dalian, China).

### Real-time quantitative polymerase chain reaction

Real-Time qPCR analysis was carried out in QuantStudio 7 Real-time fluorescent quantitative PCR system (Applied Biosystems, VA, USA). Real-time qPCR reaction was carried in a volume of 25 μL containing 16 μL Probe qPCR Mix (Code No. 391A, TaKaRa, Dalian, China), 1 μL forward primer (0.4 pmol/μL), 1 μL reverse primer (0.4 pmol/μL), 1 μL probe (0.4 pmol/μL), and 2 μL target DNA. The reaction blend was then subjected to 45 cycles at 95°C for 5 s and 60°C for 30 s, with fluorescence acquisition at each cycle. Each sample was analyzed three times.

### Specificity and cross-reactivity

The specificity and cross reactivity of the detection methods were evaluated by qPCR analysis. Undiluted sample material DNA obtained from 13 closely related different puffer fish species and 9 other unrelated fish species listed in Supplementary Table 3 was used. DNA analysis of each species was repeated no less than 3 times. All sample DNA used for the test was detected with primers and probes of internal reference 18 S rRNA to avoid inhibitory substance.

### Amplification efficiency (*E*)

In order to calculate the amplification efficiency (*E*) of qPCR, 8 series of dilution levels were prepared using the sample DNA of *Lagocephalus inermis*, *Lagocephalus lagocephalus*, *Lagocephalus gloveri*, *Lagocephalus lunaris*, and *Lagocephalus spadiceus*, including 100, 50, 20, 10, 5, 2, 1, and 0.1%. All samples were analyzed three times. Mean *Ct* values obtained for each point were plotted against the Log (DNA concentration (ng/μL), and a linear regression analysis was performed. Using the slope of the regression line, the qPCR efficiency was calculated using the equation *E* = 100 (10^–1/slope^ − 1) and expressed in percent. For each target, the slope of the regression curve should be between 3.9 and 2.9 corresponding to PCR efficiencies ranging from 80 to 120%. Additionally, the correlation coefficient *R*^2^ of the curve is a measure of the linearity of the PCR reaction. The *R*^2^ for each target should be greater than 0.98.

### Sensitivity tests

The LOD was experimentally determined according to accepted guidelines (28). DNA was extracted from *Lagocephalus inermis*, *Lagocephalus lagocephalus*, *Lagocephalus gloveri*, *Lagocephalus lunaris*, and *Lagocephalus spadiceus*, respectively, and diluted by *Limanda aspera* DNA extracted from slices of fish meat. Therefore, 8 series of dilution levels were prepared to simulate real samples for qPCR analysis and determine LOD, including: 100, 50, 20, 10, 5, 2, 1 and 0.1%. LOD_6_ of the qPCR method represents six replicate analyses performed for each dilution point of serial dilution. At least three times must be performed under repeat conditions, yielding a total of 18 results per dilution point. The lowest dilution level at which all 18 replicates show a specific positive amplification was considered as the LOD_6_. The analytical sensitivity of the qPCR method was present by LOD_95%_. The LOD_95%_ refers to the use of the corresponding LOD_6_ level, one higher dilution level, and one lower dilution level, and each level is tested 60 times. All 60 replicates showed specific positive amplification, and the lowest dilution level was considered as LOD_95%_ with a 95% confidence level. Statistical significance LOD_95%_ was calculated by Semi logarithmic regression analysis (PRISM, Graphpad Software Inc., San Diego, CA, United States), input of the corresponding number of sample materials, the number of repetitions, and the number of positive results in qPCR detection.

### Robustness evaluation

The robustness of puffer fish detection was checked by changing conditions of the qPCR reaction such as the qPCR instrument (CFX 96 Real-Time qPCR System, Bio-Rad Co., Ltd, Hercules, CA, USA), the concentration of primers and probes (±25%), and the annealing temperature S6. In each combination, a template in an amount of four times the LOD_6_ was added to the assay, at least repeat three times in one run. Regression analysis was carried out with SPSS (statistical product and service solutions, IBM Inc.) software, and the Kruskal Wallis H test was used to evaluate the significance level of difference in results obtained by orthogonal design combination of each method. When the *p* > 0.05, there was no significant difference in results.

## Results and discussion

### Development of the specific quantitative polymerase chain reaction method for five specific of puffer fish in *Lagocephalus*

**FIGURE 1:**
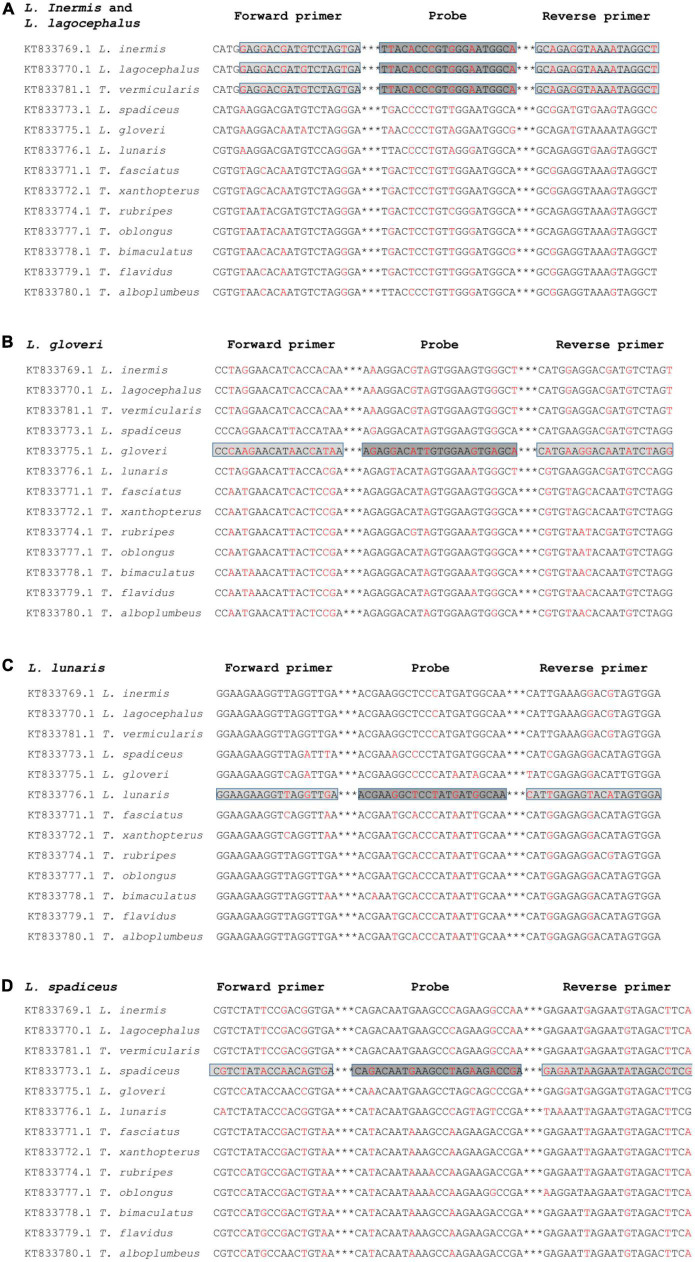
Alignment of a segment of the *COI* gene region of different puffer fish species for the development of the specific qPCR method for *L. inermis* and *L. lagocephalus* **(A)**, *L. gloveri* **(B)**, *L. lunaris* **(C)**, and *L. spadiceus* **(D)**. The location and orientation of primers and probes are indicated by lightgray and darkgray boxes, respectively. Differential bases are indicated by red. ***Represents the omitted part in oligonucleotide sequence.

The DNA fragment of the mitochondrial *Cytochrome Oxidase Sub-unit I* (*COI*) gene was selected as the target of the developed real-time qPCR method for puffer fish species identification. The base sequence of the *COI* gene region has a large genetic variation among species, but a small genetic variation within species, was stable and has high identification ability, and has been widely used for fish species identification (29–31). We have searched almost all the genus *Lagocephalus* in NCBI, there are 18 accession numbers of *COI* gene sequences of *Lagocephalus inermis* and 9 accession numbers of *COI* gene sequences of *Lagocephalus lagocephalus*. The homology of *COI* genes between these two species is 100%. In addition, there are 15 accession numbers of *Lagocephalus gloveri*, 19 accession numbers of *Lagocephalus lunaris*, and 41 accession numbers of *Lagocephalus spadiceu* in NCBI. They have differences in nucleic acid sequences, which can realize the identification of each species. The DNA sequences of 5 species of puffer fish in the genus *Lagocephalus* and 8 species of puffer fish in the genus *Takifugu* were aligned (Figure 1), and primer sequences that could distinguish the DNA sequences of 5 species of puffer fish in the genus lepidocephalus from those of other species were searched. The sequences of five *Lagocephalus* and eight *Takifugu* were comparable to search primers that can distinguish the DNA sequences of five species of *Lagocephalus*, *Takifugu* and other puffer fish species (Figure 1). All the designed primers could theoretically exclude other species of puffer fish. However, it was worth noting that the *COI* sequence of *Takifugu vermicularis* with three accession numbers in NCBI has 100% homology with that of *Lagocephalus inermis* and *Lagocephalus lagocephalus*. It is impossible to distinguish these three species based on the *COI* sequence. Nevertheless, it has been revealed that the homology analysis results of the 18 S rRNA, *Cytb*, and *COI* DNA fragment sequences of *Takifugu vermicularis* all belong to the same group as the genus *Lagocephalus*, with a homology of 99–100% (32).

### Specificity and cross-reactivity

**FIGURE 2:**
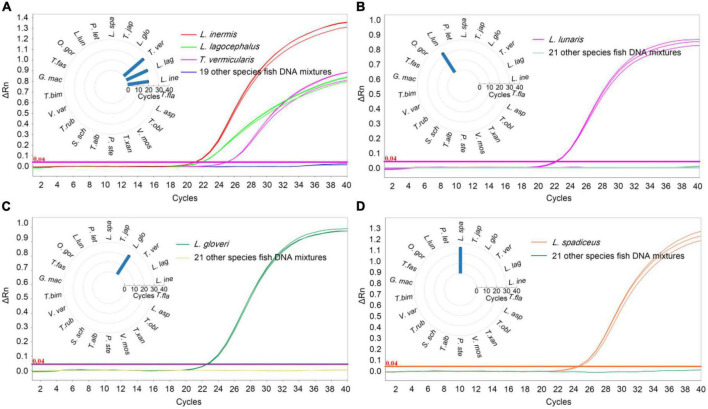
Specificity results of real-time qPCR methods. qPCR *Ct* value corresponding to puffer fish and other meat DNA, **(A)** *L. inermis*, *L. lagocephalus*, and *T. vermicularis* was detected and the *Ct* value was 20.58 ± 0.09, 22.61 ± 0.24, and 25.43 ± 0.11, respectively. Other 19 species fish were undetected. **(B)** *L. gloveri* was detected and the *Ct* value was 22.71 ± 0.38. **(C)** *L. lunaris* was detected and the *Ct* value was 22.99 ± 0.47. **(D)** *L. spadiceus* was detected and the *Ct* value was 25.98 ± 0.28. Inner: the *Ct* value for the samples with 22 species of fish. *L. ine*, *Lagocephalus inermis*; *L. lag*, *Lagocephalus lagocephalus*; *T. ver*, *Takifugu vermicularis*; *L. glo*, *Lagocephalus gloveri*; *T. jap*, *Trachurus japonicus*; *L. spa*, *Lagocephalus spadiceus*; *P. let*, *Paralichthys lethostigma*; *L. lun*, *Lagocephalus lunaris*; *O. gor*, *Oncorhynchus gorbuscha*; *T. fas*, *Takifugu fasciatus*; *G. mac*, *Gadus macrocephalus*; *T. bim*, *Takifugu bimaculatus*; *V. var*, *Verasper variegatus*; *T. rub*, *Takifugu rubripes*; *S. sch*, *Sebastes schlegelii*; *T. alb*, *Takifugu alboplumbeus*; *P. ste*, *Platichthys stellatus*; *V. mos*, *Verasper moseri*; *T. xan*, *Takifugu xanthopterus*; *T. obl*, *Takifugu oblongus*; *L. asp*, *Limanda aspera*; *T. fla*, *Takifugu flavidus*.

In order to determine the specificity of the qPCR method, the DNA of target sample materials (*L. inermis*, *L. lagocephalus*, *L. gloveri*, *L. lunaris*, and *L. spadiceus*) and non-target sample materials were analyzed. To exclude possible inhibitory effects in DNA preparations, samples were analyzed by qPCR using the primers and probe of internal reference of 18 SrRNA. All target and non-target fish species sample were successfully amplified on tested (Supplementary Table 4), confirming the suitability of sample DNA for qPCR assays. In the qPCR detection method of *L. inermis* and *L. lagocephalus*, the two species puffer fish could be detected at the same time, and had cross reactivity with the *T. vermicularis*, no false-positive signal was detected in other tested samples. The results of specificity test also verified that the sequence homology of the *COI* gene of *L. inermis*, *L. lagocephalus*, and *T. vermicularis*, indicating that the genus classification of *T. vermicularis* needs further exploration. In addition, in the respective qPCR detection methods of *L. gloveri*, *L. lunaris* and *L. spadiceus*, no cross reactivity was found in the DNA of non-target sample materials, and no false-positive signal was detected (Figure 2). These qPCR analysis results confirmed the accuracy and specificity of the detection method.

### Amplification efficiency (*E*) and linearity (*R*^2^)

**FIGURE 3:**
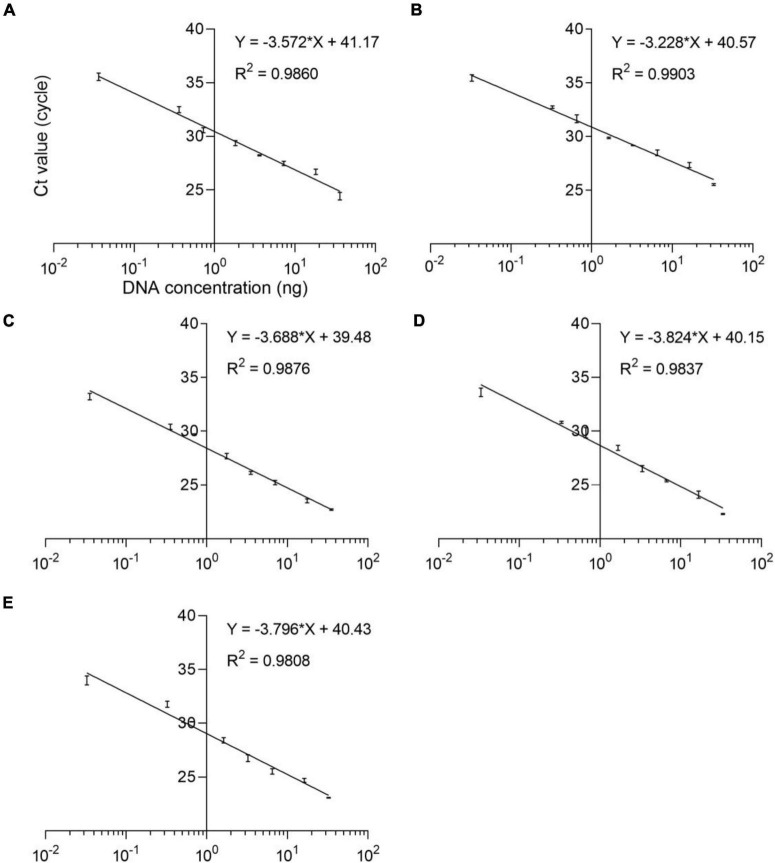
Standard curves of the analyses of eight dilution levels for the real-time qPCR assays. **(A)** *L. inermis*, **(B)** *L. lagocephalus*, **(C)** *L. gloveri*, **(D)** *L. lunaris*, **(E)** *L. spadiceus*. All samples were analyzed three times.

The qPCR efficiency of these four methods was analyzed by qPCR at eight consecutive dilution levels (*m* = 8) of DNA samples from five species puffer fish in the genus *Lagocephalus* including *L. inermis*, *L. lagocephalus*, *L. gloveri*, *L. lunaris*, and *L. spadiceus*. Each dilution level is tested at least three times. The threshold cycle value (*Ct* value) was compared with the DNA concentration (pg/μL) to draw a linear regression curve. According to the equation *E* = 100 (10^–1/slope^ − 1), the analysis efficiency is determined as the slope of the regression line, which shows a good linear relationship between *Ct* value and DNA concentration (Figure 3). The correlation coefficient *R*^2^ of these qPCR methods is 0.9808–0.9903, the slope of the regression curve is −3.824 to −3.228, and the efficiency *E* is 82.60–104.07% (Supplementary Table 5). These results match the specifications of the common qPCR validation guidelines, with a required linearity (*R*^2^) should be ≥ 0.98, the slope of the regression curve should be between −3.9 and −2.9 corresponding to an efficiency (*E*) from 80 to 120%.

### Sensitivity

**FIGURE 4:**
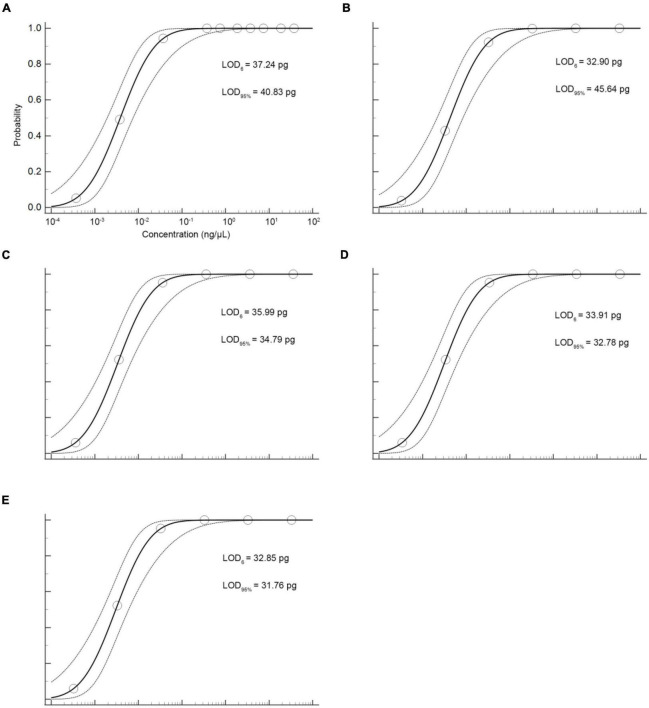
Probit regression analysis using MedCalc Software was performed on data of 6 replicates (*m* = 6, *n* = 3) from serial dilutions by the specific qPCR methods. **(A)** *L. inermis*, **(B)** *L. lagocephalus*, **(C)** *L. gloveri*, **(D)** *L. lunaris*, **(E)** *L. spadiceus*.

**FIGURE 5:**
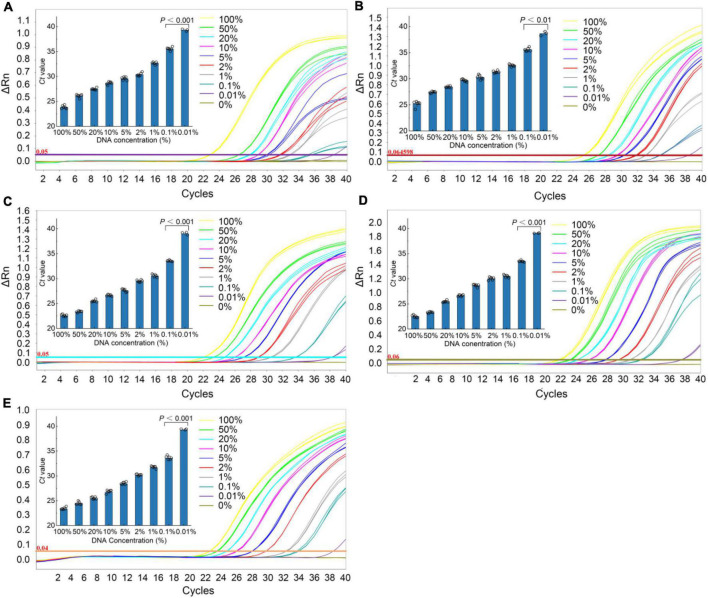
Real-time qPCR results of the sensitivity analyses. Typical fluorescence curves of qPCR method corresponding to the addition of different percentage of different species puffer fish added into mixture meat ranging from 0 to 100% (0, 0.001, 0.1, 1, 2, 5, 10, 20, 50, and 100%). Inner: the *Ct* value for the samples with 0.001–100% puffer fish. **(A)** *L. inermis*, **(B)** *L. lagocephalus*, **(C)** *L. gloveri*, **(D)** *L. lunaris*, **(E)** *L. spadiceus*. All values are presented as mean ± s.d. Statistical significances were obtained by the Mann Whitney test.

The sensitivity of the qPCR method was an important parameter that needs to be evaluated, especially considering the regulations that wild puffer fish were not allowed to eat cultured by unauthorized certification companies, and the detection of species that may contain low concentrations. In this study, the sensitivity of the qPCR LOD was expressed by LOD_6_ and LOD_95%_. It is determined by measuring the serial dilution level of DNA of five kinds of puffer fish samples in their respective detection. The LOD_6_ of *L. inermis* and *L. lagocephalus* was 37.24 pg and 32.90 pg, and the LOD_95%_ was 40.83 pg (17.31–233.44 pg, 95% CI) and 45.64 pg (19.25–265.22 pg, 95% CI), respectively. For *L. gloveri*, *L. lunaris*, and *L. spadiceus* the LOD_6_ was 35.99, 33.91, and 32.85 pg, and the LOD_95%_ was 34.79 pg (14.70–207.20 pg, 95% CI), 32.78 pg (13.85–195.23 pg, 95% CI), and 31.76 pg (13.41–189.13 pg, 95% CI), all of the five qPCR methods had highly sensitive (Supplementary Table 6 and Figure 4). This highly sensitive indicates that when the aquatic products contain 0.1% of a small amount of wild puffer fish materials without certification, they can be tracked reliably (Figure 5).

### Robustness

**FIGURE 6:**
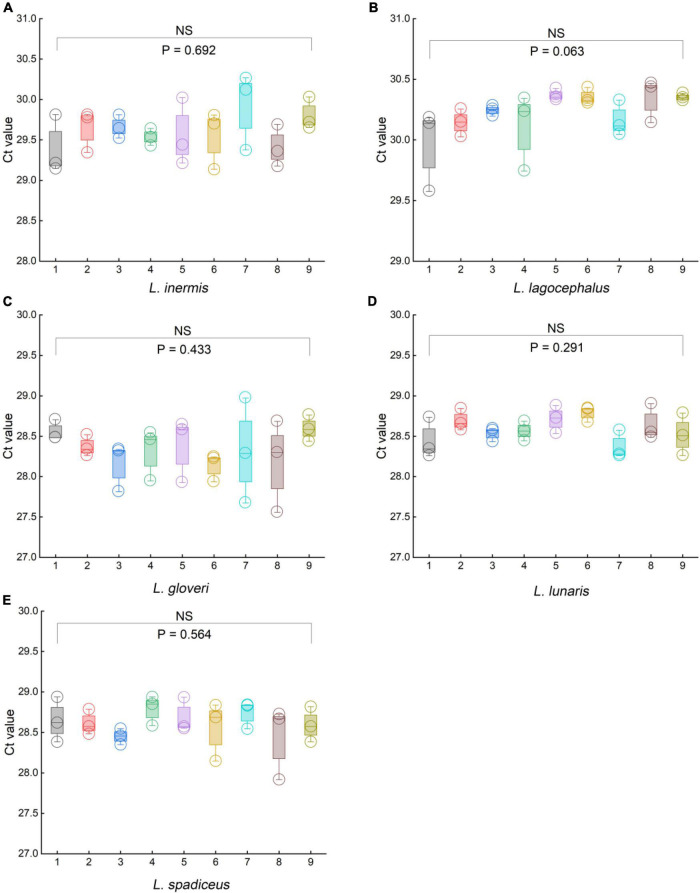
Results of the robustness experiments for the specific of qPCR methods. **(A)** *L. inermis*, **(B)** *L. lagocephalus*, **(C)** *L. gloveri*, **(D)** *L. lunaris*, **(E)** *L. spadiceus*. In **(A–E)**, box plots are centered around the median. Minima and maxima are shown as the bottom and top of the box plots, respectively. All values are presented as mean ± s.e.m. Kruskal Wallis H test was used. NS, not significant.

To evaluate the robustness of the qPCR methods, we used orthogonal design to slightly change the principal different experimental conditions, such as the qPCR instruments, qPCR reagents, primer, and probe concentrations, and slight deviations of PCR annealing temperature. We used a 5% DNA template sample to examine the impact of the aforementioned variables on the stability of the results. In the orthogonal design combination of each method, there was no significant difference in the *Ct* values of the five species of puffer fish in the genus *Lagocephalus* (Figure 6 and Supplementary Tables 7, 8). For *L. inermis*, the *Ct* value was 29.84 ± 0.43 (*p* = 0.692). For *L. lagocephalus* the *Ct* value was 30.23 ± 0.20 (*p* = 0.063). For *L. gloveri* the *Ct* value was 28.33 ± 0.34 (*p* = 0.433). For *L. lunaris* the *Ct* value was 28.59 ± 0.20 (*p* = 0.291). For *L. spadiceu*s the *Ct* value was 28.61 ± 0.24 (*p* = 0.564). Data in above are mean ± s.d. (*n* = 3). Thus, it can be concluded that the statistical *p*-value >0.05 of each method’s *Ct* values, these qPCR methods were stable, and can be transferred to other laboratories and used in routine analysis.

## Conclusion

This study describes a method for detecting five common puffer fish species belonging to the genus *Lagocephalus*: *L. inermis*, *L. lagocephalus*, *L. gloveri*, *L. lunaris*, and *L. spadiceus*. These methods were able to detect as little as 0.1% (w/w) puffer fish content, and the statistical *p*-value for each method’s *Ct* values was greater than 0.05. Each of these qPCR methods did not identify any cross-reactivity in the DNA of 21 non-target species sample materials nor detect any false-positive signals.

In summary, the developed qPCR methods were sensitive, highly specific, robust, and reproducible, which could be a viable tool for analyzing the authenticity of puffer fish aquatic goods. It is also universal, which means that it can be applied to detect any species-specific DNA sequence and thus detect other types of food fraud. This is attributed to the molecular recognition of the species-specific DNA sequences is carried out by hybridizing the analyzed DNA sequences with complementary oligonucleotide probes. This method can detect puffer fish species rapidly and end within 45 min, and also allows tracing the cause of poisoning after a food poisoning incident.

## Data availability statement

The datasets presented in this study can be found in online repositories. The names of the repository/repositories and accession number (s) can be found in the article/Supplementary material.

## Author contributions

JC, RD, and YC conceived and designed the experiments. XY and RX performed the experiments. ZL and LY analyzed the data. BH edited and wrote the manuscript. All authors contributed to the article and approved the submitted version.

## Footnotes

1
https://www.ncbi.nlm.nih.gov/genbank/

## References

[1] Bi HY Cai DD Zhang RR Zhu YW Zhang DN Qiao L Mass spectrometry-based metabolomics approach to reveal differential compounds in pufferfish soups: flavor, nutrition, and safety. *Food Chem.* (2019) 301:8. 10.1016/j.foodchem.2019.125261 31377618

[2] Xinying Yin LJ Fan M She QT You RY Lu YD Lu DC Facilely self-assembled and dual-molecule calibration aptasensor based on SERS for ultra-sensitive detection of tetrodotoxin in pufferfish. *Spectroc Acta Molec Biomolec Spectr.* (2022) 279:8. 10.1016/j.saa.2022.121275 35605417

[3] Amano M Takatani T Sakayauchi F Oi R Sakakura Y. The brain of the wild toxic marine pufferfishes accumulates tetrodotoxin. *Toxicon.* (2022) 218:1–7. 10.1016/j.toxicon.2022.08.015 36041513

[4] Zhang N Wang W Li B Liu Y. Non-volatile taste active compounds and umami evaluation in two aquacultured pufferfish (*Takifugu obscurus* and *Takifugu rubripes*). *Food Bioscience.* (2019) 32:100468. 10.1016/j.fbio.2019.100468

[5] Zhang Q Jing R Wang B Cui P Zhou C Yan T Fast mode decision based on gradient information in 3D-HEVC. *IEEE Access.* (2019) 7:135448-56.

[6] Giusti A Guarducci M Stern N Davidovich N Golani D Armani A. The importance of distinguishing pufferfish species (*Lagocephalus* spp.) in the mediterranean sea for ensuring public health: evaluation of the genetic databases reliability in supporting species identification. *Fish Res.* (2019) 210:14–21. 10.1016/j.fishres.2018.10.003

[7] Giusti A Ricci E Guarducci M Gasperetti L Davidovich N Guidi A Emerging risks in the European seafood chain: molecular identification of toxic *Lagocephalus* spp. In fresh and processed products. *Food Control.* (2018) 91:311–20. 10.1016/j.foodcont.2018.04.013

[8] Galimberti A De Mattia F Losa A Bruni I Federici S Casiraghi M DNA barcoding as a new tool for food traceability. *Food Res Int.* (2013) 50:55–63. 10.1080/07388551.2021.1874279 33530758

[9] Jurges G Sahi V Rios Rodriguez D Reich E Bhamra S Howard C Product authenticity versus globalisation-the tulsi case. *PLoS One.* (2018) 13:e0207763. 10.1371/journal.pone.0207763 30475878 PMC6261265

[10] Danezis GP Tsagkaris AS Camin F Brusic V Georgiou CA. Food authentication: techniques, trends & emerging approaches. *TrAC Trends Analy Chem.* (2016) 85:123–32. 10.1016/j.trac.2016.02.026

[11] Caira S Pinto G Nicolai MA Chianese L Addeo F. Simultaneously tracing the geographical origin and presence of bovine milk in Italian water buffalo Mozzarella cheese using MALDI-TOF data of casein signature peptides. *Anal Bioanal Chem.* (2016) 408:5609–21. 10.1007/s00216-016-9663-0 27299776

[12] Fornal E Montowska M. Species-specific peptide-based liquid chromatography-mass spectrometry monitoring of three poultry species in processed meat products. *Food Chem.* (2019) 283:489–98. 10.1016/j.foodchem.2019.01.074 30722903

[13] Stahl A Schroder U. Development of a MALDI-TOF MS-based protein fingerprint database of common food fish allowing fast and reliable identification of fraud and substitution. *J Agric Food Chem.* (2017) 65:7519–27. 10.1021/acs.jafc.7b02826 28745053

[14] Cubero-Leon E Peñalver R Maquet A. Review on metabolomics for food authentication. *Food Res Int.* (2014) 60:95–107. 10.1016/j.foodres.2013.11.041

[15] Böhme K Calo-Mata P Barros-Velázquez J Ortea I. Recent applications of omics-based technologies to main topics in food authentication. *TrAC Trends Analy Chem.* (2019) 110:221–32. 10.1016/j.trac.2018.11.005

[16] Creydt M Fischer M. Omics approaches for food authentication. *Electrophoresis.* (2018) 39:1569–81. 10.1002/elps.201800004 29572870

[17] Wu Y Yang Y Liu M Wang B Li M Chen Y. Molecular tracing of the origin of six different plant species in bee honey using real-time PCR. *J Aoac Int.* (2017) 100:744–52. 10.5740/jaoacint.16-0265 28094000

[18] Köppel R Ganeshan A Weber S Pietsch K Graf C Hochegger R Duplex digital PCR for the determination of meat proportions of sausages containing meat from chicken, turkey, horse, cow, pig and sheep. *Eur Food Res Technol.* (2019) 245:853–62.

[19] Noh ES Park YJ Kim EM Park JY Shim KB Choi TJ Quantitative analysis of alaska pollock in seafood products by droplet digital PCR. *Food Chem.* (2019) 275:638–43. 10.1016/j.foodchem.2018.09.093 30724244

[20] Wu YY Liu M Wang B Han J Chen Y. A 15-plex/xMAP method to detect 15 animal ingredients by suspension array system coupled with multifluorescent magnetic beads. *J Aoac Int.* (2016). [Epub ahead of print]. 10.5740/jaoacint.15-0216 27098447

[21] Haynes E Jimenez E Pardo MA Helyar SJ. The future of NGS (next generation sequencing) analysis in testing food authenticity. *Food Control.* (2019) 101:134–43. 10.3390/foods11081108 35454695 PMC9027865

[22] Manikandan M. DNA as a biomaterial in diagnosis of food adulteration and food safety assurance. *Res Dev Material Sci.* (2017) 2:3.

[23] Ali ME Amin MA Razzak MA Abd Hamid SB Rahman MM Abdul Rashid N Short amplicon-length PCR assay targeting mitochondrial cytochrome b gene for the detection of feline meats in burger formulation. *Food Analy Methods.* (2015) 9:571–81.

[24] Doosti A Ghasemi Dehkordi P Rahimi E. Molecular assay to fraud identification of meat products. *J Food Sci Technol.* (2014) 51:148–52. 10.1007/s13197-011-0456-3 24426061 PMC3857419

[25] Marieschi M Torelli A Beghe D Bruni R. Authentication of *Punica granatum* L.: development of SCAR markers for the detection of 10 fruits potentially used in economically motivated adulteration. *Food Chem.* (2016) 202:438–44. 10.1016/j.foodchem.2016.02.011 26920316

[26] Schiefenhovel K Rehbein H. Differentiation of sparidae species by DNA sequence analysis. PCR SSCP and IEF of sarcoplasmic proteins. *Food Chem.* (2013) 138:154–60. 10.1016/j.foodchem.2012.10.057 23265470

[27] Tamura SKGSaK. MEGA7: molecular evolutionary genetics analysis version 7.0 for bigger datasets. *Mol Biol Evol.* (2016) 33:1870–4. 10.1093/molbev/msw054 27004904 PMC8210823

[28] Broeders S Huber I Grohmann L Berben G Taverniers I Mazzara M Guidelines for validation of qualitative real-time PCR methods. *Trends Food Sci Technol.* (2014) 37:115–26. 10.1016/j.tifs.2014.03.008

[29] Asis AM Lacsamana JK Santos MD. Illegal trade of regulated and protected aquatic species in the philippines detected by DNA barcoding. *Mitochondrial DNA DNA Mapp Seq Anal.* (2016) 27:659–66. 10.3109/19401736.2014.913138 24841434

[30] Goncalves PF Oliveira-Marques AR Matsumoto TE Miyaki CY. DNA barcoding identifies illegal parrot trade. *J Hered.* (2015) 106:560–4. 10.1093/jhered/esv035 26245790

[31] Stein FM Wong JCY Sheng V Law CSW Schröder B Baker DM. Erratum to: first genetic evidence of illegal trade in endangered European eel (anguilla anguilla) from Europe to Asia. *Conservat Genet Res.* (2016) 8:539.

[32] Wenbing Chen TM Weng G Chen R Shao B Peng J Jiang S. Analysis of partial DNA sequence of COI gene and its application for species identification of thirteen species pufferfish. *Chin Food Sci.* (2018) 39:145–8.

