# Supplementary material for: Real-time qPCR for the detection of puffer fish components from Lagocephalus in food: L. inermis, L. lagocephalus, L. gloveri, L. lunaris, and L. spadiceus
Source: Front Nutr. 2022 Dec 5;9:1068767. doi: 10.3389/fnut.2022.1068767 (PMC9760932; doi:10.3389/fnut.2022.1068767)
Supplement: Supplementary file 1 [file Data_Sheet_1.docx]

Supplementary Material

**Supplementary Table 1.** All fish samples used in this study

| Name | Latin name |
| --- | --- |
| Smooth blaasop | *Lagocephalus inermis* |
| Oceanic puffer | *Lagocephalus lagocephalus* |
| Glover´s puffer | *Lagocephalus gloveri* |
| Lunartail puffer | *Lagocephalus lunaris* |
| Silver-cheeked toadfish | *Lagocephalus spadiceus* |
| Purple puffer | *Takifugu vermicularis* |
| Obscura puffer | *Takifugu fasciatus* |
| Yellowfin puffer | *Takifugu xanthopterus* |
| Twospotpuffer | *Takifugu bimaculatus* |
| Tawny Puffer | *Takifugu flavidus* |
| Tiger Puffer | *Takifugu rubripes* |
| Lattice blaasop | *Takifugu oblongus* |
| White spot puffer | *Takifugu alboplumbeus* |
| Yellowfin sole | *Limanda aspera* |
| Variegate flounder | *Verasper variegatus* |
| barfm flounder | *Verasper moseri* |
| starry flounder | *Platichthys stellatus* |
| southern flounder | *Paralichthys lethostigma* |
| Humpback | *Oncorhynchus gorbuscha* |
| Pacific hake | *Gadus macrocephalus* |
| Korean rockfish | *Sebastes schlegelii* |
| Japanese Jack Mackerel | *Trachurus japonicus* |

**Supplementary Table 2.** Accession Numbers of *cytochrome oxidase subunit I* (*COI*) gene used for Real-Time PCR Assays

| Species | Accession number | *COI* gene size (bp) | Species | Accession number | *COI* gene size (bp) |
| --- | --- | --- | --- | --- | --- |
| *L. inermis* | KT833769.1 | 681 | *L. lunaris* | MF588660.1 | 559 |
| *L. inermis* | GU674214.1 | 648 | *L. lunaris* | MF588653.1 | 547 |
| *L. inermis* | GU674212.1 | 648 | *L. lunaris* | LC155442.1 | 636 |
| *L. inermis* | GU674211.1 | 648 | *L. lunaris* | KU945261.1 | 552 |
| *L. inermis* | GU674209.1 | 648 | *L. lunaris* | KU945251.1 | 552 |
| *L. inermis* | LC155441.1 | 636 | *L. lunaris* | KU945236.1 | 552 |
| *L. inermis* | KU945263.1 | 552 | *L. lunaris* | FJ434550.1 | 1546 |
| *L. inermis* | KU945262.1 | 552 | *L. lunaris* | JQ681799.1 | 677 |
| *L. inermis* | KU893011.1 | 552 | *L. spadiceus* | KT833773.1 | 681 |
| *L. inermis* | FJ434549.1 | 1546 | *L. spadiceus* | GU674445.1 | 648 |
| *L. inermis* | JF493725.1 | 652 | *L. spadiceus* | GU674243.1 | 652 |
| *L. inermis* | GU804920.1 | 648 | *L. spadiceus* | JN312835.1 | 652 |
| *L. inermis* | JX995946.1 | 614 | *L. spadiceus* | HQ564335.1 | 648 |
| *L. inermis* | JX995945.1 | 614 | *L. spadiceus* | KR861536.1 | 649 |
| *L. inermis* | JX995944.1 | 614 | *L. spadiceus* | KR861535.1 | 649 |
| *L. inermis* | JX995943.1 | 614 | *L. spadiceus* | KM538381.1 | 663 |
| *L. inermis* | JX995942.1 | 614 | *L. spadiceus* | KM538380.1 | 663 |
| *L. inermis* | KP266776.1 | 655 | *L. spadiceus* | KM538379.1 | 663 |
| *L. lagocephalus* | KT833770.1 | 681 | *L. spadiceus* | KM538378.1 | 663 |
| *L. lagocephalus* | KX586199.1 | 652 | *L. spadiceus* | KM538377.1 | 663 |
| *L. lagocephalus* | MG559740.1 | 597 | *L. spadiceus* | KM538376.1 | 663 |
| *L. lagocephalus* | JF730882.1 | 655 | *L. spadiceus* | KM538375.1 | 663 |
| *L. lagocephalus* | JF730881.1 | 655 | *L. spadiceus* | KM538374.1 | 663 |
| *L. lagocephalus* | MH638677.1 | 676 | *L. spadiceus* | KM538373.1 | 663 |
| *L. lagocephalus* | LC155440.1 | 636 | *L. spadiceus* | KM538372.1 | 663 |
| *L. lagocephalus* | JQ681798.1 | 677 | *L. spadiceus* | KM538371.1 | 663 |
| *L. lagocephalus* | HQ010074.1 | 582 | *L. spadiceus* | KM538370.1 | 663 |
| *L. gloveri* | KT833775.1 | 681 | *L. spadiceus* | KM538369.1 | 663 |
| *L. gloveri* | KY984986.1 | 707 | *L. spadiceus* | KM538368.1 | 663 |
| *L. gloveri* | LC155437.1 | 636 | *L. spadiceus* | KM538367.1 | 663 |
| *L. gloveri* | LC155436.1 | 636 | *L. spadiceus* | KM538366.1 | 663 |
| *L. gloveri* | KU945235.1 | 552 | *L. spadiceus* | KM538365.1 | 663 |
| *L. gloveri* | KU893012.1 | 525 | *L. spadiceus* | KY130423.1 | 704 |
| *L. gloveri* | FJ434548.1 | 1546 | *L. spadiceus* | MK777842.1 | 488 |
| *L. gloveri* | KP641409.1 | 568 | *L. spadiceus* | MK777841.1 | 525 |
| *L. gloveri* | KP641408.1 | 568 | *L. spadiceus* | MK777840.1 | 508 |
| *L. gloveri* | KP641407.1 | 568 | *L. spadiceus* | MK777839.1 | 451 |
| *L. gloveri* | KP641406.1 | 568 | *L. spadiceus* | MK777838.1 | 608 |
| *L. gloveri* | KP641405.1 | 568 | *L. spadiceus* | LC155438.1 | 636 |
| *L. gloveri* | KP641404.1 | 568 | *L. spadiceus* | EU595163.1 | 652 |
| *L. gloveri* | KP641403.1 | 568 | *L. spadiceus* | EU595162.1 | 652 |
| *L. gloveri* | KP641402.1 | 568 | *L. spadiceus* | EU595161.1 | 652 |
| *L. lunaris* | KT833776.1 | 681 | *L. spadiceus* | EU595160.1 | 652 |
| *L. lunaris* | MW498661.1 | 654 | *L. spadiceus* | EF607419.1 | 652 |
| *L. lunaris* | MW498660.1 | 654 | *L. spadiceus* | KT718614.1 | 621 |
| *L. lunaris* | MW498659.1 | 654 | *L. spadiceus* | KT718613.1 | 616 |
| *L. lunaris* | MW498658.1 | 654 | *L. spadiceus* | HQ167726.1 | 652 |
| *L. lunaris* | MW498657.1 | 654 | *L. spadiceus* | FJ384711.1 | 445 |
| *L. lunaris* | KF489625.1 | 652 | *L. spadiceus* | KP266858.1 | 655 |
| *L. lunaris* | MN511929.1 | 655 | *T. vermicularis* | KT833781.1 | 681 |
| *L. lunaris* | MK331954.1 | 668 | *T. vermicularis* | FJ434559.1 | 1546 |
| *L. lunaris* | MH429329.1 | 623 | *T. vermicularis* | KY514079.1 | 682 |
| *L. lunaris* | MF588661.1 | 546 |  |  |  |

**Supplementary Table 3.** Primers and Probes Used in This Study ^a^

|  | primer and probe sequence (5′→ 3′) | Genbank No. | position | size (bp) |
| --- | --- | --- | --- | --- |
| *L. Inermis* and *L. lagocephalus* | | | | |
| forward primer | GAGGACGATGTCTAGTGA | KT833769.1  KT833770.1 | 254-271 | 196 |
| reverse primer | AGCCTATTTTACCTCTGC |  | 431-449 |  |
| probe | FAM-TGCCATTCCCACGGGTGTAA-BHQ1 |  | 399-419 |  |
| *L. gloveri* | | | | |
| forward primer | CCCAAGAACATAACCATAA | KT833775.1 | 96-114 | 174 |
| reverse primer | CCTAGATATTGTCCTTCATG |  | 249-269 |  |
| probe | FAM-TGCTCACTTCCACAATGTCCTCT-BHQ1 |  | 211-233 |  |
| *L. lunaris* | | | | |
| forward primer | GGAAGAAGGTTAGGTTGA | KT833776.1 | 76-93 | 150 |
| reverse primer | TCCACTATGTACTCTCAATG |  | 206-225 |  |
| probe | FAM-TTGCCATCATAGGAGCCTTCGT-BHQ1 |  | 174-195 |  |
| *L. spadiceus* | | | | |
| forward primer | CGTCTATACCAACAGTGA | KT833773.1 | 450-467 | 173 |
| reverse primer | CGAGGTCTATATTCTTATTCTC |  | 611-632 |  |
| probe | FAM-CAGACAATGAAGCCTAGAAGACCGA-BHQ1 |  | 491-516 |  |
| 18SrRNA | | | | |
| forward primer | TCTGCCCTATCAACTTTCGATGGTA | XR3887404.1 | 337-361 | 137 |
| reverse primer | AATTTGCGCGCCTGCTGCCTTCCTT |  | 449-473 |  |
| probe | FAM-CCGTTTCTCAGGCTCCCTCTCCGGAATCGAACC- BHQ1 |  | 405-437 |  |
| ^a^ FAM, 6-carboxy-fluorescein; BHQ1, black hole quencher1. | | | | |

**Supplementary Table 4.** Real-Time qPCR Results of the Specificity Analyses

| Common name | Latin name | Average *Ct* value of qPCR assays | | | | |
| --- | --- | --- | --- | --- | --- | --- |
|  |  | *L. Inermis* and *L. lagocephalus* | *L. gloveri* | *L. lunaris* | *L. lunaris* | 18SrRNA |
| Smooth blaasop | *L. inermis* | 20.58 ± 0.09 | negative | negative | negative | 14.53 ± 0.05 |
| Oceanic puffer | *L. lagocephalus* | 22.61 ± 0.24 | negative | negative | negative | 14.89 ± 0.04 |
| Glover´s puffer | *L. gloveri* | negative | 22.71 ± 0.38 | negative | negative | 14.67 ± 0.04 |
| Lunartail puffer | *L. lunaris* | negative | negative | 22.99 ± 0.47 | negative | 14.24 ± 0.04 |
| Silver-cheeked toadfish | *L. spadiceus* | negative | negative | negative | 25.98 ± 0.28 | 14.19 ± 0.03 |
| Purple puffer | *T. vermicularis* | 25.43 ± 0.11 | negative | negative | negative | 14.52 ± 0.04 |
| Obscure puffer | *T. fasciatus* | negative | negative | negative | negative | 14.41 ± 0.03 |
| Yellowfin puffer | *T. xanthopterus* | negative | negative | negative | negative | 14.55 ± 0.06 |
| Two spot puffer | *T. bimaculatus* | negative | negative | negative | negative | 14.32 ± 0.03 |
| Tawny Puffer | *T. flavidus* | negative | negative | negative | negative | 14.13 ± 0.03 |
| Tiger Puffer | *T. rubripes* | negative | negative | negative | negative | 14.79 ± 0.04 |
| Lattice blaasop | *T. oblongus* | negative | negative | negative | negative | 14.51 ± 0.03 |
| White spot puffer | *T. alboplumbeus* | negative | negative | negative | negative | 15.52 ± 0.06 |
| Yellowfin Sole | *Limanda aspera* | negative | negative | negative | negative | 14.56 ± 0.04 |
| Variegate flounder | *Verasper variegatus* | negative | negative | negative | negative | 14.37 ± 0.03 |
| barfm flounder | *Verasper moseri* | negative | negative | negative | negative | 14.82 ± 0.04 |
| starry flounder | *Platichthys stellatus* | negative | negative | negative | negative | 14.75 ± 0.03 |
| southern flounder | *Paralichthys lethostigma* | negative | negative | negative | negative | 14.29 ± 0.03 |
| Humpback | *Oncorhynchus gorbuscha* | negative | negative | negative | negative | 14.11 ± 0.03 |
| Pacific hake | *Gadus macrocephalus* | negative | negative | negative | negative | 14.54 ± 0.04 |
| Korean rockfish | *Sebastes schlegelii* | negative | negative | negative | negative | 14.36 ± 0.04 |
| Japanese Jack Mackerel | *Trachurus japonicus* | negative | negative | negative | negative | 14.45 ± 0.03 |

**Supplementary Table 5.** Amplification Efficiency (*E*) and Linearity (*R*^2^) for the qPCR Assays (*R*^2^) ^a^

| assays | linear regression equation | slope | efficiency (*E*) | Linearity(*R*^2^) |
| --- | --- | --- | --- | --- |
| *L. inermis* | Y = -3.572*X + 41.17 | -3.572 | 90.53% | 0.9860 |
| *L. lagocephalus* | Y = -3.228*X + 40.57 | -3.228 | 104.07% | 0.9903 |
| *L. gloveri* | Y = -3.688*X + 39.48 | -3.688 | 86.70% | 0.9876 |
| *L. lunaris* | Y = -3.824*X + 40.15 | -3.824 | 82.60% | 0.9837 |
| *L. spadiceus* | Y = -3.796*X + 40.43 | -3.796 | 83.42% | 0.9808 |
| ^a^Y, the plotted *Ct* value. X, the DNA concentration. | | | | |

**Supplementary Table 6.** LOD_6_ and LOD_95%_ for the Specific of qPCR Assays

| Species | LOD_6_ | | LOD_95%_ | |
| --- | --- | --- | --- | --- |
|  | percentage, % | DNA concentration, pg | DNA concentration, pg | 95% CI |
| *L. inermis* | 0.1 | 37.24 | 40.83 | 17.31-233.44 |
| *L. lagocephalus* | 0.1 | 32.90 | 45.64 | 19.25-265.22 |
| *L. gloveri* | 0.1 | 35.99 | 34.79 | 14.70-207.20 |
| *L. lunaris* | 0.1 | 33.91 | 32.78 | 13.85-195.23 |
| *L. spadiceus* | 0.1 | 32.85 | 31.76 | 13.41-189.13 |

**Supplementary Table 7.** Results of the Robustness Experiments for the specific of qPCR methods

| Species | 1 | 2 | 3 | 4 | 5 | 6 | 7 | 8 | 9 |  |
| --- | --- | --- | --- | --- | --- | --- | --- | --- | --- | --- |
| *L. inermis* | | 29.39±0.37 | 29.65±0.26 | 29.66±0.14 | 29.54±0.11 | 29.56±0.42 | 29.55±0.36 | 29.92±0.48 | 29.41±0.26 | 29.80±0.20 |
| *L. lagocephalus* | | 29.96±0.34 | 30.14±0.11 | 30.24±0.04 | 30.11±0.32 | 30.37±0.05 | 30.35±0.07 | 30.16±0.14 | 30.35±0.18 | 30.35±0.03 |
| *L. gloveri* | | 28.56±0.13 | 28.37±0.13 | 28.15±0.30 | 28.31±0.32 | 28.38±0.40 | 28.13±0.17 | 28.31±0.65 | 28.18±0.57 | 28.59±0.16 |
| *L. lunaris* | | 28.45±0.25 | 28.69±0.14 | 28.53±0.09 | 28.56±0.12 | 28.71±0.17 | 28.79±0.10 | 28.37±0.18 | 28.65±0.22 | 28.58±0.18 |
| *L. spadiceus* | | 28.65±0.28 | 28.61±0.16 | 28.45±0.10 | 28.79±0.18 | 28.68±0.21 | 28.56±0.36 | 28.74±0.17 | 28.44±0.45 | 28.59±0.22 |

**Supplementary Table 8.** Conditions of the Robustness Experiments as an Orthogonal Design ^a^

| Factors | Combination | | | | | | | | |
| --- | --- | --- | --- | --- | --- | --- | --- | --- | --- |
|  | 1 | 2 | 3 | 4 | 5 | 6 | 7 | 8 | 9 |
| qPCR instrument | A | × | × | × | × | × | A | × | × |
| qPCR reagents | B | B | × | × | × | × | B | × | × |
| primer concentration (pmol/μL) | × | × | 0.3 | 0.5 | × | × | × | × | × |
| probe concentration (pmol/μL) | × | × | × | × | 0.3 | 0.5 | × | × | × |
| annealing temperature | × | × | × | × | × | × | +1℃ | −1℃ | × |
| ^a^ ×, standard conditions; A, CFX96, Bio-Rad,Hercules, CA, U.S.A.; B, GoTaq qPCR Master Mix,CodeA6101, Promega Co., Ltd. USA | | | | | | | | | |
